# Therapeutic Effect of CAOLD Chemotherapy Regimen on Patients With Relapsed/Refractory Angioimmunoblastic T-Cell Lymphoma: A Case Study

**DOI:** 10.3389/fonc.2021.758445

**Published:** 2022-01-03

**Authors:** Yu Liu, Pingping Li, Liren Qian

**Affiliations:** ^1^ Senior Department of Hematology, The Fifth Medical Center of PLA General Hospital, Beijing, China; ^2^ Department of Hematology, The Sixth Medical Center of PLA General Hospital, Beijing, China; ^3^ Clinical Laboratory Department of Bejing Boren Hospital, Beijing, China

**Keywords:** peripheral T-cell lymphoma, chemotherapy, angioimmunoblastic T-cell lymphoma, CAOLD, CHOP

## Abstract

Angioimmunoblastic T-cell lymphoma (AITL) is a kind of peripheral T-cell lymphomas (PTCLs) with a highly invasive feature. At present, patients are often treated with CHOP or CHOP-like regimens which is of poor prognosis whilst having high recurrence rate. Once the patient fails to achieve remission or relapse after the first-line treatment, many salvage chemotherapy regimens are always ineffective, and the long-term survival will be difficult to achieve for them. In this circumstance, more effective therapy methods are needed. In this study, two patients with relapsed/refractory AITL were treated with the CAOLD regimen [cyclophosphamide 400 mg/m^2^ qd d1, cytarabine 30 mg/m^2^ qd d1–d4, vindesine 2 mg/m^2^ qd d1, pegaspargase (PEG-ASP) 2,500 IU/m^2^ qd d2, dexamethasone 7.5 mg/m^2^ qd d1–d5], and long-term remission was achieved after chemotherapy. One is still alive after achieving complete remission (CR) after two cycles of chemotherapy, who has been followed up for 82 months. Besides, another patient achieved partial remission (PR) after the first course of chemotherapy. Then, CR was obtained after four courses of consolidation chemotherapy. The patient has been followed up for 63 months and is still alive. Both of them achieved long-time survival. These two successful cases demonstrated that the CAOLD regimen can be a better choice for relapsed/refractory AITL and offers hope of breakthrough in this medical field.

## Introduction

Angioimmunoblastic T-cell lymphoma (AITL) accounts for 1%–2% of non-Hodgkin’s lymphoma and 18.5% of peripheral T-cell lymphomas (PTCLs) (1), which includes hemolytic anemia, systemic lymphadenopathy, B symptoms, and hepatosplenomegaly as its main clinical manifestations (2). Besides, aggregation of atypia small lymphocytes around endothelial microvascular, active proliferation of eosinophils and plasma cells, and expression of CXCL13 and PD-1 in malignant cells are the main pathological features of AITL (3). The NCCN Guidelines suggest that all patients with AITL should participate in clinical trials, indicating that there is no definite treatment plan for AITL currently. CHOP (cyclophosphamide, adriamycin, vincristine, prednisone) and CHOPE (CHOP plus etoposide) were often selected as first-line chemotherapy in most of the AITL cases. The prognosis of AITL is poor as many cases relapsed after complete remission (CR) (4). Moreover, the recurrence rate of AITL is about 56%, and the median survival period takes 36 months, while the survival rate reaches 36% in the past 5 years (3). Nowadays, various targeted therapeutic drugs combined with chemotherapy were still in the clinical trial stage for relapsed/refractory AITL, such as alemtuzumab, belinostat, romidepsin, and bortezomib. Thus, the second-line treatment also brings us great challenges.

We found that L-Asp may take effect on AITL after consulting relevant data and literature, whether it was a single drug treatment or combined with other regimens. Asparaginase is an enzyme preparation from *Escherichia coli*, which can hydrolyze asparagine in serum into aspartic acid and ammonia. Additionally, asparagine is an essential amino acid for cells synthesizing proteins and for proliferation. In general, normal cells can synthesize asparagine, but this function does not work in tumor cells. Therefore, we used asparaginase combined with cyclophosphamide, cytarabine, vindesine, and dexamethasone for these two patients with relapsed/refractory AITL, and finally it implies a good therapeutic effect.

## Case Presentation

The two patients were diagnosed as AITL by pathological examination. The disease stage was determined according to the Ann Arbor classification (5), and the International Prognostic Index was also calculated (6).

### Patient 1

**Figure 1:**
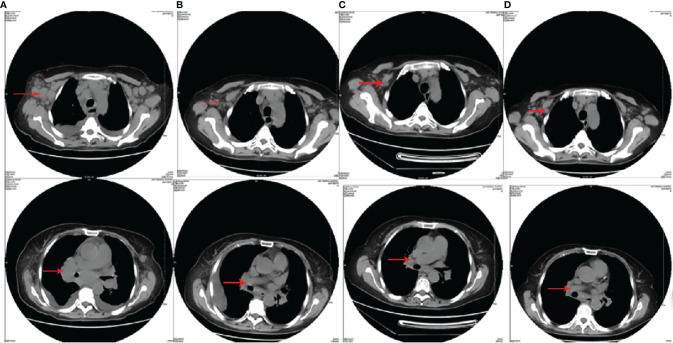
The chest CT images of patient 1. **(A)** The chest CT before CHOPE (bilateral axillary, mediastinum, bilateral hilar, and bilateral cervical lymphonodus were enlarged); **(B)** the chest CT after three cycles of CHOPE, before CAOLD (mediastinal and hilar lymphonodus enlarged again; the size of axillary lymphonodus was the same as before); **(C)** the chest CT after two cycles of CAOLD (mediastinal and hilar lymphonodus were significantly reduced); **(D)** the chest CT after five cycles of CAOLD (mediastinal and hilar enlarged lymphonodus almost disappeared).

A 63-year-old woman was hospitalized in her local hospital due to chest distress and shortness of breath. The result of chest CT test showed that her right hilar space was occupied by unknown substances, accompanied by mediastinal lymph node enlargement and bilateral pleural effusion. Moreover, abnormal increase of FDG metabolism in extensive lymph nodes, spleen, and right lobe of the liver was found *via* PET-CT. These were considered as malignant lesions. However, biopsy of the lymph nodes in the left neck and armpit showed reactive hyperplasia. In order to further clarify the diagnosis, the patient transferred to our hospital in October 2014. We found that the patient had a low respiratory sound in both lungs, and the chest CT examination showed bilateral pleural effusion. The left axillary pathological tissue of the patient was examined by pathological experts, thus confirming the pathological diagnosis. The result of the immunohistochemistry included the proliferative heterotypic lymphocytes CD3(+++), CD2(+++), CD5(+++), CD4(+++), BCL-6(++), CXCL13(++), minority CD10(+), and Ki-67 (30%); proliferative immunoblast CD30(+) and CD15(+); plasmocyte CD138(+), IgG(+), and IgG4(−); minority T-cell CD7(+),CD8(+), and GrB(+); residual lymphoid follicles CD20(++), CD21(++), BCL-2(++), and CD38(++); histocyte CD68(+); and proliferative vascular endothelial cells CD34(+). The pathological experts agreed that the pathological characteristics and immunohistochemistry of the left axillary lymph nodes were consistent with AITL and EBER(+). Moreover, the cytological and pathological examination of pleural effusion was also consistent with AITL. Combined with the symptoms, signs, and examination results of the patient, AITL was comprehensively diagnosed (Ann Arbor classification was stage IV, IPI 3). The first cycle of chemotherapy by CHOPE (cyclophosphamide, doxorubicin, vincristine, prednisone, etoposide) was given in October 2014, and partial remission (PR) was evaluated after chemotherapy. Then, after two cycles of CHOPE consolidation, the chest CT was re-examined, and progression of the disease was indicated. In January 2015, she received chemotherapy by CAOLD regimens. The patient refused to get examined by PET-CT and CR was achieved after two cycles of CAOLD by CT (**Figure 1**). There were no obvious side effects during the treatment and the patient was consolidated with another three cycles of treatment. The physical condition of this selected case has been monitored throughout 82 months by our team and she is alive so far.

### Patient 2

**Figure 2:**
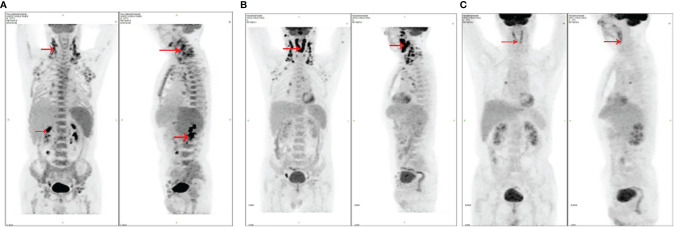
The PET-CT images of patient 2. **(A)** PET-CT before chemotherapy (systemic multiple lymphadenopathy with FDG hypermetabolism); **(B)** PET-CT after three cycles of GEMOX, before CAOLD (multiple enlarged lymphonodus, abnormally high-glucose metabolism, some new lesions, such as left anterior ear, right parotid gland, bilateral oropharynx, and parapharyngeal); **(C)** PET-CT after four cycles of CAOLD (the enlarged lymphonodus and glucose metabolism disappeared).

A 76-year-old male, who had a 1-month history of recurrent fever, received treatment in our hospital in 2016. At that time, his enlarged lymph nodes can be touched evidently behind the ear, neck, supraclavicular, and axillary. Moreover, many lymph nodes in the right neck were swollen on ultrasound examination with the largest being the size of 3.2 * 1.2 cm. Subsequently, the lymph nodes were surgically removed, and as a result, the pathological features and immunohistochemical results are consistent with AITL and EBER(+). The result of the immunohistochemistry was composed of tumor cell LCA(+++), CD2(+++), CD3(+++), CD5(+++), CD7(+++), CD4(+++), CD8(+), CD20(−), CXCL-13(−), PD-1(++), minority CD10(+), BCL-2(++), BLC-6(+++), ALK(−), P53(−), and Ki-67 (40%) and immunoblastic CD30(+) and EMA(+). Moreover, transabdominal ultrasound showed enlargement of the liver and spleen, while bone marrow puncture showed no involvement in bone marrow morphology. On the other hand, it was notable that the IgM of the EB virus antibody in the plasma was 46 U/ml (normal range should be 0–20 U/ml), the nucleic acid quantification of the EB virus (plasma) was 3,410 copies (normal level should be less than 1,000 copies), and the nucleic acid quantification of the EB virus (PBMC) was 2,894,000 copies (normal level should be lower than 1,000 copies). From this perspective, combining with the result of PET-CT, it can be seen that lymphoma invaded extensively the lymph nodes and spleen. Taking into consideration the symptoms, signs, and examination results of the patient, AITL was comprehensively diagnosed (Ann Arbor classification as stage IV, IPI 3). Thereafter, he received GEMOX chemotherapy (gemcitabine and oxaliplatin) on April 21, 2016. After three cycles of chemotherapy, progressive enlargement of cervical lymph nodes was developed and PET-CT indicated the progression of lymphoma. In this case, the CAOLD chemotherapy was given on July 2016. Specifically, he achieved CR after four cycles of chemotherapy, which was evaluated by PET-CT (**Figure 2**). In the end, the EB virus in the blood turned negative, and the size of the spleen returned to normal. It is worth noting that during the first, third, and fifth cycles of chemotherapy, the patient had developed severe neutrophil deficiency, followed by bacterial infection, which was easily cured by antibiotics. The treatment was stopped after six cycles of chemotherapy. This patient has been followed up for 63 months and he is still alive so far with CR.

## Discussion

AITL is a kind of malignant lymphoma and lymph node biopsy is required for the diagnosis of AITL. Currently, the treatment of AITL is still diversified. CHOP or CHOP-like regimens have always been the first-line chemotherapy. The total OR and CR rate of the patients treated with CHOP are 70% to 79% and 35% to 39%, respectively (7–9). However, the recurrence rate is still high (8). The data of each center shows that the 5-year progression-free survival (PFS) rate of patients treated with CHOP is only 18%–20% (10). In a retrospective study of 199 patients with PTCL in British Columbia Cancer Agency (9), the CR rate of the patients with AITL treated with the CHOP regimen was 70%; however, the 5-year overall survival (OS) and PFS were 36% and 13%, respectively. Although patients treated with the CHOP regimen achieved a high remission rate, their recurrence rate is high and the level of PFS is low. Other first-line chemotherapy options include CHOPE and dose-adjusted EPOCH. In a prospective study, CHOP and CHOPE were compared on 28 AITL younger patients (11). The 3-year EFS rate in the CHOPE group was significantly higher than that in the CHOP group (67.5% *vs.* 50%). Nevertheless, there was no difference in terms of the 3-year OS rate. Other drugs combined with CHOP, such as alemtuzumab (12), belinostat (13), romidepsin (14), and bortezomib (15), improved the CR rate to a certain extent, while the side effects also increased. In addition, the combination of brentuximab vedotin (BV) and CHP has been recommended as the first-line treatment. Brentuximab vedotin is a CD30 antibody drug conjugate. CD30 is generally expressed in systemic anaplastic large cell lymphoma. It is also partially expressed in PTCL, among which 43%–63% of AITL patients expressed CD30 (16). In a phase 3 global trial that is double-blinded and randomized (17), the 5-year PFS and OS in the BV plus CHP group were significantly better than those in the CHOP group, and there were no significant differences in adverse effects between the two groups. However, most of the above new drugs are still in clinical trials and have not been widely used in the clinic; hence, there is no recommended standard chemotherapy for refractory/relapsed AITL patients.

L-asparaginase has been demonstrated to have an excellent effect in *in vitro* anti-NK-cell lymphoma activity (18). In an international phase II study of the L-asparaginase-containing regimen SMILE (19), an ORR of 79% was observed in relapsed and refractory patients with advanced-stage disease. Based on these encouraging results, another international study examined the use of SMILE with sandwiched radiotherapy in 29 stage I/II patients (20). After the initial two to three cycles of SMILE, the ORR and CR rate were 86.2 and 69%. Thus, L-asparaginase is very important for nasal NK/T-cell lymphoma. Also, few reports have shown that asparaginase can be effective on PTCL. A retrospective study analyzed 102 patients with incipient PTCL who received L-asparaginase (administered as 6,000 U/m^2^/day, for 7 days) with or without multidrug chemotherapy regimens. Patients who received L-asparaginase containing multidrug chemotherapy had higher OR rate than those who received L-asparaginase-free ones (83.3% *vs.* 61.7%, *P* = 0.016), particularly those at phase III/IV (82.4% *vs.* 54.0%, *P* = 0.007) and with an International Prognostic Index (IPI) score of ≥2 (82.1% *vs.* 50.0%, *P* = 0.006) (21). It shows that asparaginase containing multidrug chemotherapy regimen in incipient PTCL showed a better short-term effect and controllable adverse effects. Tsutomu et al. presented a relapsed patient with Epstein–Barr virus (EBV)-positive cytotoxic PTCL-NOS, who was successfully treated using L-ASP alone. L-ASP treatment was initiated at 6,000 U/m^2^ on days 1, 3, 5, 10, and 12 together with prednisolone at a dose of 1 mg/kg. Although he developed grade 2 liver dysfunction and grade 2 coagulopathy, the patient achieved CR (22). In view of these studies, we added sufficient dosage of asparaginase to the CAOLD protocol.

Cytarabine is rare in chemotherapy regimens for peripheral T lymphoma. For example, the ESHAP regimen contains high-dose cytarabine. In this retrospective study, ESHAP (etoposide, methylprednisolone, high-dose Ara-C, and cisplatin) offers a long-term survival in some transplant ineligible patients with PTCLs who were chemosensitive with late relapse after frontline therapy (23). From January 2005 to April 2015, 33 patients with R/R PTCLs received ESHAP as first salvage regimen at Chiang Mai University Hospital. The overall response rate was 46% (CR 39%). The median duration of response was 18 months. Median second PFS and OS were 8.0 and 11.0 months, respectively. Patients having late relapse had more favorable OS than those having early relapsed or refractory disease with a median OS of 21, 17, and 3 months, respectively (*P* = 0.001). Patients achieving CR after ESHAP had significantly better median OS (39, 7, and 5 months, *P* < 0.0001) and second PFS (33, 2, and 2 months, *P* < 0.0001) than those achieving PR or having progressive disease. Grade 3–4 neutropenia (45.5%) and thrombocytopenia (33.4%) were common but manageable. It can be seen that high-dose cytarabine has a certain curative effect in R/R peripheral T-cell lymphoma.

The dosage of all drugs was selected according to the actual situation of the patients. Compared with the previous chemotherapy regimen, the biggest advantage of this regimen is the addition of asparaginase. So, a sufficient dosage of asparaginase (2,500 IU/m^2^) was given. The dosage of other drugs was reduced because of old age and poor physical condition. We reduced the original dosage of cyclophosphamide and dexamethasone almost by half, the dosage of cyclophosphamide was reduced from 750 to 400 mg/m^2^, and the dosage of dexamethasone was reduced from 15 mg/m^2^ (equivalent to 100 mg/m^2^ prednisone) to 7.5 mg/m^2^. The dosage of vindesine was also reduced from 3 to 2 mg/m^2^. Because we have added sufficient ASP on the basis of cyclophosphamide, vindesine, and dexamethasone, adding high-dose cytarabine may cause uncontrollable side effects. So, we decided to start with low-dose cytarabine (30 mg/m^2^) and gradually increase the dose. Our first concern of this scheme was its safety. We were also worried that it will not achieve effective therapeutic effect. However, when the CAOLD regimen was used in the first patient, the efficacy was significant, so we did not increase the dosage of other drugs. The duration of the free interval between cycles was 28 days. We still recommend a cycle every 21 days if the patient tolerated it well and has no obvious side effects. Simultaneously, there were no serious side effects. The main advantage of this choice is to increase a certain curative effect and avoid some uncontrollable side effects. This scheme should not be selected for patients with varying degrees of liver function injury before treatment or in previous treatment because of ASP. Because of the low incidence rate, there are still few large prospective studies on relapse/refractory AITL. Most clinicians chose a treatment plan according to clinical experience and the actual situation of the patients. In this study, we reported two patients with relapse/refractory AITL who were successfully treated with CAOLD. Both of them obtained long-term continuous CR, and no fatal side effect appeared. Its promising result provides a new method to treat patients with relapse/refractory AITL.

To sum up, these two successful cases showed that the CAOLD regimen may be a better choice for AITL, which provides the foundation for future large sample size clinical trial. Because of the low incidence rate of these two selected cases, our team was unable to collect further cases, so we were limited in making effective statistical significance as there should be more case samples to verify its effectiveness. Besides, basic research studies are also expected to explore the mechanism under the therapeutic effects.

## Data Availability Statement

The original contributions presented in the study are included in the article/supplementary material. Further inquiries can be directed to the corresponding author.

## Ethics Statement

The studies involving human participants were reviewed and approved by Committee of the Sixth Medical Center of Chinese PLA General Hospital. The patients/participants provided their written informed consent to participate in this study. Written informed consent was obtained from the individual(s) for the publication of any potentially identifiable images or data included in this article.

## Author Contributions

YL and PL collected the data and wrote the manuscript. LQ designed the research. All authors contributed to the article and approved the submitted version.

## Funding

This work was supported by a grant from the National Defense Science and Technology Innovation Special Zone Project-Spark Project (Grant No. 20-163-00-TS-009-006-01) and a grant from the National Natural Science Foundation of China (Grant No. 81800180).

## Conflict of Interest

The authors declare that the research was conducted in the absence of any commercial or financial relationships that could be construed as a potential conflict of interest.

## Publisher’s Note

All claims expressed in this article are solely those of the authors and do not necessarily represent those of their affiliated organizations, or those of the publisher, the editors and the reviewers. Any product that may be evaluated in this article, or claim that may be made by its manufacturer, is not guaranteed or endorsed by the publisher.

## References

[1] Federico M Rudiger T Bellei M Nathwani BN Luminari S Coiffier B. Clinicopathologic Characteristics of Angioimmunoblastic T-Cell Lymphoma: Analysis of the International Peripheral T-Cell Lymphoma Project. J Clin Oncol (2013) 31:240–6. doi: 10.1200/JCO.2011.37.3647 PMC3532394 22869878

[2] Lunning MA Vose JM. Angioimmunoblastic T-Cell Lymphoma: The Many-Faced Lymphoma. Blood (2017) 129:1095–102. doi: 10.1182/blood-2016-09-692541 28115369

[3] Broccoli A Zinzani PL. Angioimmunoblastic T-Cell Lymphoma. Hematol Oncol Clin North Am (2017) 31:223–38. doi: 10.1016/j.hoc.2016.12.001 28340875

[4] Moskowitz AJ. Practical Treatment Approach for Angioimmunoblastic T-Cell Lymphoma. J Oncol Pract (2019) 15:137–43. doi: 10.1200/JOP.18.00511 PMC7850668 30861367

[5] Carbone PP Kaplan HS Musshoff K Smithers DW Tubiana M. Report of the Committee on Hodgkin’s Disease Staging Classification. Cancer Res (1971) 31:1860–1. 5121694

[6] P. International Non-Hodgkin’s Lymphoma Prognostic Factors. A Predictive Model for Aggressive non-Hodgkin’s Lymphoma. N Engl J Med (1993) 329:987–94. doi: 10.1056/NEJM199309303291402 8141877

[7] Reimer P Rudiger T Geissinger E Weissinger F Nerl C Schmitz N. Autologous Stem-Cell Transplantation as First-Line Therapy in Peripheral T-Cell Lymphomas: Results of a Prospective Multicenter Study. J Clin Oncol (2009) 27:106–13. doi: 10.1200/JCO.2008.17.4870 19029417

[8] Simon A Peoch M Casassus P Deconinck E Colombat P Desablens B. Upfront VIP-Reinforced-ABVD (VIP-rABVD) is Not Superior to CHOP/21 in Newly Diagnosed Peripheral T Cell Lymphoma. Results of the Randomized Phase III Trial GOELAMS-Ltp95. Br J Haematol (2010) 151:159–66. doi: 10.1111/j.1365-2141.2010.08329.x 20738307

[9] Savage KJ Chhanabhai M Gascoyne RD Connors JM. Characterization of Peripheral T-Cell Lymphomas in a Single North American Institution by the WHO Classification. Ann Oncol (2004) 15:1467–75. doi: 10.1093/annonc/mdh392 15367405

[10] Ellin F Landstrom J Jerkeman M Relander T. Real-World Data on Prognostic Factors and Treatment in Peripheral T-Cell Lymphomas: A Study From the Swedish Lymphoma Registry. Blood (2014) 124:1570–7. doi: 10.1182/blood-2014-04-573089 25006130

[11] Schmitz N Trumper L Ziepert M Nickelsen M Ho AD Metzner B. Treatment and Prognosis of Mature T-Cell and NK-Cell Lymphoma: An Analysis of Patients With T-Cell Lymphoma Treated in Studies of the German High-Grade Non-Hodgkin Lymphoma Study Group. Blood (2010) 116:3418–25. doi: 10.1182/blood-2010-02-270785 20660290

[12] Gallamini A Zaja F Patti C Billio A Specchia MR Tucci A. Alemtuzumab (Campath-1H) and CHOP Chemotherapy as First-Line Treatment of Peripheral T-Cell Lymphoma: Results of a GITIL (Gruppo Italiano Terapie Innovative Nei Linfomi) Prospective Multicenter Trial. Blood (2007) 110:2316–23. doi: 10.1182/blood-2007-02-074641 17581918

[13] Knauf WU Koenigsmann MP Notter M Hoppe B Reufi B Oberberg D. Peripheral Blood Progenitor Cell Mobilization With Dexa-Beam/G-CSF, Ether Lipid Purging, and Autologous Transplantation After High-Dose CBV Treatment: A Safe and Effective Regimen in Patients With Poor Risk Malignant Lymphomas. Leuk Lymphoma (1996) 23:305–11. doi: 10.3109/10428199609054833 9031111

[14] Dupuis J Morschhauser F Ghesquieres H Tilly H Casasnovas O Thieblemont C. Combination of Romidepsin With Cyclophosphamide, Doxorubicin, Vincristine, and Prednisone in Previously Untreated Patients With Peripheral T-Cell Lymphoma: A non-Randomised, Phase 1b/2 Study. Lancet Haematol (2015) 2:e160–5. doi: 10.1016/S2352-3026(15)00023-X 26687958

[15] Kim SJ Yoon DH Kang HJ Kim JS Park SK Kim HJ. Bortezomib in Combination With CHOP as First-Line Treatment for Patients With Stage III/IV Peripheral T-Cell Lymphomas: A Multicentre, Single-Arm, Phase 2 Trial. Eur J Cancer (2012) 48:3223–31. doi: 10.1016/j.ejca.2012.06.003 22770877

[16] Sabattini E Pizzi M Tabanelli V Baldin P Sacchetti CS Agostinelli C. CD30 Expression in Peripheral T-Cell Lymphomas. Haematologica (2013) 98:e81–2. doi: 10.3324/haematol.2013.084913 PMC3729886 23716537

[17] Horwitz S O’Connor OA Pro B Illidge T Fanale M Advani R. Brentuximab Vedotin With Chemotherapy for CD30-Positive Peripheral T-Cell Lymphoma (ECHELON-2): A Global, Double-Blind, Randomised, Phase 3 Trial. Lancet (2019) 393:229–40. doi: 10.1016/S0140-6736(18)32984-2 PMC6436818 30522922

[18] Ando M Sugimoto K Kitoh T Sasaki M Mukai K Ando J. Selective Apoptosis of Natural Killer-Cell Tumours by L-Asparaginase. Br J Haematol (2005) 130:860–8. doi: 10.1111/j.1365-2141.2005.05694.x 16156856

[19] Yamaguchi M Kwong YL Kim WS Maeda Y Hashimoto C Suh C. Phase II Study of SMILE Chemotherapy for Newly Diagnosed Stage IV, Relapsed, or Refractory Extranodal Natural Killer (NK)/T-Cell Lymphoma, Nasal Type: The NK-Cell Tumor Study Group Study. J Clin Oncol (2011) 29:4410–6. doi: 10.1200/JCO.2011.35.6287 21990393

[20] Kwong YL Kim WS Lim ST Kim SJ Tang T Tse E. SMILE for Natural Killer/T-Cell Lymphoma: Analysis of Safety and Efficacy From the Asia Lymphoma Study Group. Blood (2012) 120:2973–80. doi: 10.1182/blood-2012-05-431460 22919026

[21] Yao G Zhou D Zhou M Bao C He D Li L. Clinical Analysis and Prognostic Significance of L-Asparaginase Containing Multidrug Chemotherapy Regimen in Incipient Peripheral T-Cell Lymphoma. Int J Clin Exp Med (2015) 8:9374–83. PMC4537992 26309599

[22] Takahashi T Ikejiri F Onishi C Kawakami K Inoue M Miyake T. L-Asparaginase-Induced Complete Response in a Relapsed Patient With Epstein-Barr Virus and Cytotoxic Peripheral T-Cell Lymphoma Not Otherwise Specified. Intern Med (2010) 49:2505–8. doi: 10.2169/internalmedicine.49.4083 21088358

[23] Norasetthada L Tantiworawit A Rattanathammethee T Chai-Adisaksopha C Chaipoh T Rattarittamrong E. Efficacy of ESHAP Regimen in Transplant Ineligible Patients With Relapsed/Refractory T-Cell Lymphoma. J Hematol (2018) 7:131–9. doi: 10.14740/jh459w PMC7155849 32300428

